# Predictors of seasonal influenza vaccination in chronic asthma

**DOI:** 10.1186/2049-6958-8-68

**Published:** 2013-10-21

**Authors:** Rachelle Asciak, Martin Balzan, Jesmar Buttigieg

**Affiliations:** 1 Department of Medicine, Mater Dei Hospital, 211, Street Margaret Street Tal Qroqq, Msida MSD 2090, Malta

**Keywords:** Asthma, Influenza, Vaccine

## Abstract

**Background:**

Guidelines advise annual influenza vaccination in chronic asthma. The aim of this study was to determine uptake of the influenza vaccine in a group of patients (n = 146) with moderate to severe chronic asthma and establish the main predictors of vaccination.

**Method:**

Patients attending a hospital asthma clinic were asked to complete a questionnaire in February 2012 (n = 146). These same patients were contacted a year later via telephone (n = 109 responded), and they were asked to complete the same questionnaire.

**Results:**

Vaccination rate was 50.3% in winter 2011/12, and 57.8% in 2012/13. Using binary logistic regression, the predictors for vaccination in 2012 were patient advice (Odds ratio [OR] 15.37 p = 0.001), female gender (OR 2.75, p = 0.028), past side effects (OR 0.21, p = 0.001) and comorbidity (OR 0.39, p = 0.013). Stepwise regression resulted in age as predictor (T value = 3.99, p = 0.001). On analyzing the responses from the second questionnaire at one year after attendance to asthma clinic, predictors changed to compliance to medication (OR 9.52, p= 0.001) and previous exacerbations (OR 4.19, p = 0.026). Out of the 56 patients vaccinated in 2011/12, 33 reported asthma exacerbations before 2012, and 29 reported asthma exacerbations after receiving the influenza vaccine. Out of the 46 unvaccinated patients in 2012, 27 had asthma exacerbations before 2012 and 19 patients had exacerbations in 2013. Patients vaccinated in 2011/12 needed 0.59 courses of steroid/patient/year, and 1.23 visits for nebulizer/patient/year while non-vaccinated patients needed 0.18 courses of steroids/patient/year (p = 0.048), and 0.65 visits for nebulized/patient/year (p = 0.012). Patients’ subjective statements broadly confirmed the predictors. 16/69 (23.1%) received the vaccine in winter 2012/13 despite reporting previous side effects.

**Conclusions:**

Advice to patient, female gender and patients’ age predicted vaccination, while past side effects to the influenza vaccine, and presence of comorbidities predicted non vaccination. Symptomatic asthma patients are more likely to be vaccinated. One year after the first contact, treatment compliance and previous asthma exacerbations gained statistical significance as predictors of vaccination.

## Background

Evidence shows that influenza vaccination leads to decreased hospitalization from influenza complications, fewer deaths during the influenza season, decreased healthcare costs in the elderly in the general population [1], and a decreased number of lost workdays and physician visits in healthy adults [2]. Asthma patients are considered to be at increased risk of influenza complications, however there is conflicting evidence on the beneficial effect of influenza vaccination on asthma exacerbations. Some studies suggest that, at least in children, the vaccine decreases asthma exacerbations [3], however in adults, a meta-analysis of published studies has failed to demonstrate a significant decrease in asthma exacerbations [4].

Despite this lack of evidence, the Global Initiative for Asthma (GINA) guidelines issued in December 2011, advise that patients with moderate to severe asthma should receive influenza vaccination every year or at least when vaccination of the general population is advised. The British Thoracic Society (BTS) guidelines on asthma 2008, revised in June 2009, also advise administration of the influenza vaccine independent of any considerations related to asthma. The Advisory Committee on Immunization Practices (ACIP) recommends annual influenza vaccination for adults and children with chronic disorders of the pulmonary or cardiovascular systems, including asthma.

This study was performed at Mater Dei Hospital, the University hospital of Malta (population c. 411,277). At this hospital the asthma clinics are run by Consultant Respiratory physicians, who together with specialist trainees in respiratory medicine and general medicine, see to patients attending these clinics. The clinics are run with the help of a nurse. Many asthma patients are followed up in the community, however most of the more severe cases are referred to the asthma clinic for follow up.

The aim of this study was to determine the uptake of the influenza vaccine in a group of patients with moderate to severe chronic asthma and to try to establish the main predictors of vaccination.

## Method

Between 17^th^ January and 18^th^ February 2012, adult patients with chronic asthma attending a hospital asthma clinic were asked to fill in a standardized questionnaire, with a Maltese and English version available according to patients’ preference. Data collected included age, gender, asthma control, whether patients had been advised to receive the influenza vaccine or not, and if so by whom. Questions included information on previous vaccination and side effects to the vaccine, whether the patient had received the vaccine this year or not, and the reasons behind his/her decision. Patients were also asked about comorbidities such as chronic kidney disease, for which guidelines also advise influenza vaccination. Asthma control was assessed by frequency of salbutamol use or reliever medication, the need for systemic steroids or nebulized treatment, and hospitalization over the past 12 months. After patients had filled in the questionnaire, participants received advice on the importance of influenza vaccination in asthma patients.

The following year, in February 2013, the same patients were contacted by telephone and the same questionnaire was used, in order to follow up and compare results with the previous year. At least two attempts were made to contact each patient.

Patients were considered to have well-controlled asthma if they rarely or never required their reliever salbutamol inhaler, and did not need hospitalization, oral steroids or nebulized treatment over the previous year. They were considered to have poorly controlled asthma if they used salbutamol several times daily and/or needed hospitalization, oral steroids and nebulized treatment during the previous year.

Compliance was assessed by asking patients how often they forgot to take their medication. Those who never or rarely forgot to take their asthma treatment were defined as compliant, while patients who forgot to take their treatment more than once a week or on a daily basis were defined as being non-compliant. Patients with incomplete questionnaires were excluded from the study.

### Statistical analysis

The data collected was analyzed using Microsoft Office Access® and Excel®. Categorical data was summarized using percentages, and Fisher’s two-tailed exact test was used for categorical values. Binary logistic regression and Stepwise regression was determined using Minitab 16 software. P <0.05 was considered to be statistically significant.

### Consent

Authorization to perform this study was obtained from the hospital’s data protection officer. Data protection approval was deemed sufficient from an ethical point of view. Consent was obtained from all the Consultant Respiratory physicians in the hospital to interview asthma patients under their care. Consent to perform the questionnaires was obtained from all patients.

## Results

A total of 146 patients (103 females, 43 males, mean age 47.9 years SD 19.3) suffering from chronic asthma were studied. Out of these patients, 80 individuals (63 females and 17 males) received the influenza vaccine during the winter of 2011/12. This is a 50.3% vaccination rate after correcting for gender.

109 patients (78 female, 31 male; mean age 53.6, SD 18.0) responded to the second questionnaire the following year, and vaccination rate rose to 57.8% (M 45.2%, F 70.5%) after correcting for gender.

Table 1 shows the characteristics of the original 146 patients by gender. The mean age for those taking the vaccine was 55.06 years, while the mean age for those not taking the vaccine was 42.77 years (p = 0.0002). 86.2% of the patients had been advised to take the vaccine. 62.4% of the patients who were advised to take the vaccine were vaccinated in winter 2011/12, while only 10% of those not advised were vaccinated that winter (p = <0.0001).

**Table 1:** Characteristics of patients by gender in the 2011/12 questionnaire

**Descriptor**	**Male ( *n *** **= 43)**	**Female ( *n *** **= 103)**	**Averaged rate**
Mean age	47.95	50.41	
Years of asthma	18.34	15.44	
Life-time non-smokers	65.12%	79.61%	72.36%
Ex smoker	27.91%	16.50%	22.21%
Current smoker	6.98%	3.88%	5.43%
Reliever use			
Never	41.86%	42.57%	42.22%
≤ twice weekly	6.98%	9.90%	8.44%
> twice weekly	4.65%	19.80%	12.23%
Once daily	16.28%	8.91%	12.59%
> once daily	30.23%	18.81%	24.52%
Compliant to preventer therapy	80.95%	73.74%	77.34%
Exacerbations previous year			
Exacerbation occurrence	51.16%	59.22%	55.19%
Nebulizer therapy	34.88%	43.69%	39.29%
Oral steroid administration	32.56%	35.92%	34.24%
Hospitalization for asthma	13.95%	14.56%	14.26%
Intensive care admission	0.00%	2.91%	1.46%
Comorbidity	26.19%	30.30%	28.25%
Hypertension	23.26%	27.18%	25.22%
Diabetes	11.63%	10.68%	11.15%
Ischaemic heart disease	4.65%	2.91%	3.78%
Chronic kidney disease	2.33%	0.97%	1.65%

**Figure 1:**
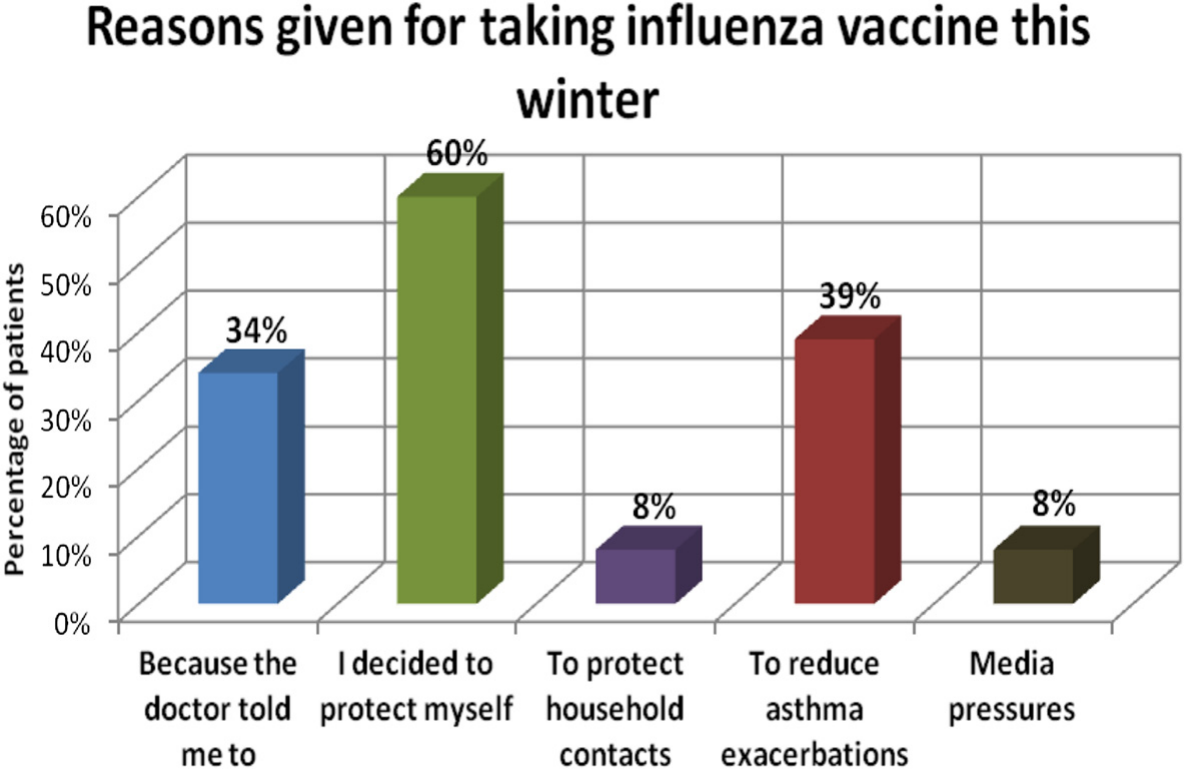
Reasons asthma patients gave for taking influenza vaccine in winter 2011/12.

**Figure 2:**
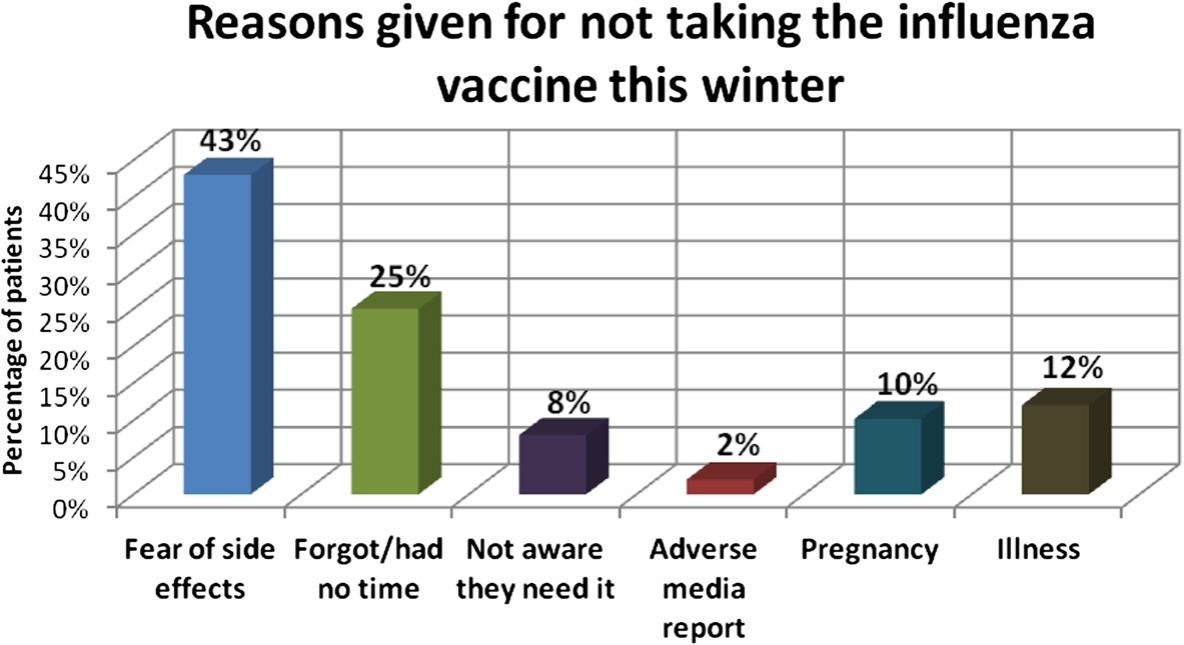
Reasons given by patients for not taking influenza vaccine in winter.

In Table 2, binary logistic regression and stepwise regression show patient advice and female gender as predictors for taking the vaccine, while comorbidity and a history of previous side effects to the vaccine are shown to be the main negative predictors for vaccination. Figure 1 shows the reasons asthma patients gave for taking the influenza vaccine that winter. Figure 2 shows the reasons given by the patients for not taking the vaccine (patients were given the option of choosing more than one reason).

**Table 2:** **Binary logistic regression and stepwise regression of possible factors predicting vaccination for influenza in 2011/12 (*n *=146)**

**Predictor (binary logistic regression)**	***p***	**Odds ratio**	**95% CI**	
Advised to take vaccine	0.001	15.37	2.98	79.13
Female gender	0.028	2.75	1.11	6.8
Compliance to medication	0.278	1.69	0.65	4.37
Exacerbations in previous year	0.294	1.61	0.66	3.9
Patient age	0.01	1.05	1.02	1.08
Asthma years	0.265	1.02	0.99	1.05
Frequency of reliever use	0.263	0.86	0.65	1.12
Comorbidity (0–4)	0.013	0.39	0.19	0.82
Previous side effects	0.001	0.21	0.08	0.53
**Stepwise regression**	*p*	T value		
Advised to take vaccine	0.001	4.15		
Previous side effects	0.001	−3.54		
Patient age	0.001	3.99		
Comorbidity (0–4)	0.021	−2.34		
Female gender	0.021	2.34		

With regard to the 2012/13 period, 92 out of 109 (84.4%) patients who responded to the questionnaire in 2013 reported previous vaccination, 78 (71.6%) had received the vaccine at least once over the previous two years, while 51 (46.8%) received the vaccine in both years. 18 out of the 109 patients (17%) received the vaccine in winter 2012/13 but not in 2011/12. On the other hand 9/109 patients did not revaccinate in 2012/13. Predictors of vaccination in 2012/13 using logistic and stepwise regression are listed in Table 3 (102 patients with complete data utilized).

**Table 3:** Odds ratio for predictors of vaccination in 2012/13 (n = 102)

**Binary logistic regression**	***p***	**Odds ratio**	**95% CI**	
Compliance to medication	0.001	9.52	2.42	37.5
Exacerbation in last year	0.018	4.8	1.31	17.6
Female gender	0.026	4.19	1.19	14.77
Advised to vaccinate	0.124	3.22	0.72	14.31
Frequency of reliever use	0.125	1.35	0.92	1.97
Age	0.017	1.05	1.01	1.09
Number of years asthma	0.232	0.98	0.94	1.02
Comorbidity (0–4)	0.575	0.66	0.16	2.81
Previous side effects	0.001	0.08	0.02	0.3
Stepwise regression	*p*	T Value		
Compliance to medication	0.001	3.4		
Previous side effects	0.001	−4.14		
Exacerbation past year	0.011	2.6		
Age	0.021	2.34		
Female gender	0.022	2.33		

According to the 51 patients who received the vaccine in both years, the reasons for re-vaccination in 2012/13 were doctor’s advice (66.7%), to protect themselves (39.2%), to protect family (13.7%), and to prevent asthma exacerbations (47.1%). The reasons given for vaccination in 2012/13 by patients who had not received the influenza vaccine the previous year (n = 18) were physician advice 17 (93.5%,p = 0.002), to protect themselves - 5 (27.8%), to protect family 1 (5.6%), and to prevent asthma exacerbations 4 (22.2%, p = 0.039).

31 patients (28.4%) did not receive the influenza vaccine in either year. Out of these, 22 (71.0%) said this was because of fear of vaccine side effects. On the other hand, there were 7 patients who had received the vaccine in winter 2011/12, but not in 2012/13, and the reasons given were fear of vaccine side effects (2), patients forgot to take the vaccine (4), and patients did not feel the need to take the vaccine (1).

13 out of 51 (25.5%) patients vaccinated in both years, and 16 out of 69 (23.1%) patients vaccinated in 2012/13 had received the influenza vaccine in the second consecutive year despite reporting previous side effects to the vaccine.

Asthma reliever medication use amongst patients vaccinated in 2012/13, *n* = 69, (no information available on two subjects), showed that 20 (29.0%) used short-acting beta agonist daily or more frequently, 19 (27.5%) used it 2-6 times per week, and 30 (43.5%) never used it. For non vaccinated patients in 2012/13 (n = 40) data was 17.5%, 22.5%, 60.0%, respectively (p = 0.22).

33 (58.9%) out of 56 patients vaccinated in 2011/12 reported asthma exacerbations in the year preceding vaccination, and 29 reported exacerbations in the year following vaccination, while out of 46 unvaccinated patients, 27 reported exacerbations in the year prior to vaccination compared to 19 in the following year.

Patients vaccinated in 2011/12 needed 0.59 courses of systemic steroids/patient/year, and 1.23 visits for nebulizer/patient/year in the following year. Non vaccinated patients needed 0.18 courses of systemic steroids/patient/year (p = 0.048), and 0.65 visits for nebulizer/patient/year (p = 0.012) respectively. Unfortunately data prior to vaccination was not available.

## Discussion

This study showed that despite the recommendation for influenza vaccination in asthma guidelines, just under half of the patients with asthma failed to take the influenza vaccine. The decision whether to take it or not is influenced by several factors including trust or mistrust in modern medicine, perceived side-effects from prior vaccination, perceived risk associated with influenza [5], and concern that vaccination may induce exacerbations of asthma. A randomized controlled trial (RCT) involving 2,032 adults with asthma [6], and a large multicentre cohort study [7] concluded that influenza vaccination in asthma is safe.

In our study, the reported influenza vaccination rate of 57.8% was encouraging when compared to the 39.9% for adult asthmatic patients in 2006-2007 in the USA [8], and 40% vaccination rate in asthma patients in 2003 in a single urban British general practice in Exeter [9].

This study was carried out on patients attending a hospital asthma clinic; therefore patients were likely to suffer from more severe asthma compared to patients treated in primary care. In fact, 55.19% reported exacerbations in the previous year, and 77.34% reported to be prescribed and to be compliant with preventer medication.

The power of this study was insufficient to gauge the impact of vaccination as there was an insignificant difference in exacerbation rates before and after vaccination for individual vaccinated patients. However, there was a significantly higher asthma exacerbation rate in vaccinated patients when compared to unvaccinated patients. Though not reaching statistical significance, non-vaccinated patients seemed to use more short-acting reliever medication. This could reflect the possibility that patients with more severe symptoms are more likely to vaccinate against influenza. On the contrary, it is also possible that those patients with less severe or more intermittent asthma are less likely to vaccinate. Furthermore, perhaps more reliance on reliever medication reflected a different behavioural attitude which was not otherwise assessed in this study.

Unvaccinated patients reported a lower number of exacerbations in the second year. This was probably the result of new attendants to the asthma clinic who besides being offered vaccination could have their asthma better controlled with medication. While this reason is speculative, this explanation is supported by the emergence of compliance to medication and the occurrence of previous exacerbations as a predictor for vaccination after one year of attendance to the asthma clinic.

Using binary logistic regression in the first questionnaire, advice to the patient, and patient gender were the best predictors of vaccination, while presence of comorbidities and having experienced previous vaccine side effects were the negative predictors.

The effect of gender on vaccination rates in the general population varies significantly between countries [10], and in July 2010 a publication by the World Health Organization in entitled ‘Sex, gender and influenza’ states that the severity of asthma tends to be worse in women than in men, and women are more likely than men to be caregivers. Because of this, it is possible that women could be more aware of influenza risk and the necessity to vaccinate themselves.

Other factors have been shown to affect influenza vaccination in asthma, including increased vaccination rates with age [11]. In this study while the mean age of vaccinated patients was higher than in non-vaccinated patients, logistic regression failed to show it as a predictor, while stepwise regression re-instated it. This may have occurred because the stepwise regression model used only 5 statistically significant predictors out of the 9 predictors evaluated, removing the possible confounders of age. Logistic regression had evaluated all the 9 predictors.

In the first questionnaire, receiving advice to vaccinate heavily predicted influenza vaccination, but this effect was greatly decreased in the second questionnaire as probably many patients with a mindset not to vaccinate had also received advice during the first questionnaire, thus leveling off the difference between vaccinated and non vaccinated individuals. However, one year after attendance to the asthma clinic the second questionnaire produced two new predictors, namely: compliance to medication and occurrence of previous exacerbations. This indicates that overall, patients at the asthma clinic were not only advised to receive the influenza vaccine, but their asthma treatment was also optimized.

18 patients who were vaccinated in 2012/13 and not in 2011/12 nearly unanimously stated that advice was crucial in their decision making, especially doctors’ advice. Indeed all patients on direct questioning highly rated the impact of physician advice on their decision to vaccinate. Medical literature confirms that physician recommendations and education about influenza vaccine availability, effectiveness, and adverse effects [12] were other factors which were found to influence parents to vaccinate their asthmatic children.

Both logistic regression and stepwise regression showed that the most consistent negative predictor was the occurrence of past side effects to the influenza vaccine. This was confirmed as the main reason for not vaccinating by the patients’ responses to direct questioning. Notwithstanding this, a significant proportion of patients do actually vaccinate despite having experienced previous vaccine side effects. This may reflect that side effects are often minor when compared to the fear of major attacks of acute asthma. This reason is supported by the fact there was a greater likelihood for severely affected patients to vaccinate.

Public Health vaccination campaigns tend to target mainly the elderly population, who are more likely to suffer from comorbidities. However in this study despite the fact that 28% of patients had comorbidities, logistic and stepwise regression gave a negative predictive value. The study cannot offer an explanation for this result, except that this may be a possible reason for an increased fear of side effects of vaccination.

The main limitations of the study were that information on whether the patient had been vaccinated or not during the previous 12 months was obtained by self-report. The statistical power was too low to give an indication of the potential benefit of vaccination on the control of asthma symptoms. Furthermore, the hospital environment might have influenced respondents to praise the effect of medical advice and perhaps overestimate their compliance to medication.

## Conclusions

Advice by a medical practitioner or health care professional, female gender of patients, and increasing patient age resulted in a higher vaccination rate. Fear of side-effects and the presence of comorbidities were negative predictors of vaccination. While this study could not assess the effectiveness of the vaccine in preventing severe attacks, patients with more severe symptoms are more likely to vaccinate against influenza. One year after the first questionnaire, compliance to medication and occurrence of a previous exacerbation became a positive predictor of vaccination. Around a fourth of patients with asthma still vaccinate against influenza despite the previous occurrence of side effects.

## Competing interest

The authors declare that they have no competing interest.

## Authors’ contributions

RA study design, obtaining consent from Consultant physicians and hospital data protection office, data collection, inputting and analysis, drafting and revision of manuscript. JB Data inputting and data analysis. MB study design, supervision of the study, data analysis, statistics, drafting and revision of manuscript. All authors read and approved the final manuscript.
